# Validation of IMU against optical reference and development of open-source pipeline: proof of concept case report in a participant with transfemoral amputation fitted with a Percutaneous Osseointegrated Implant

**DOI:** 10.1186/s12984-024-01426-6

**Published:** 2024-07-31

**Authors:** Kirstin Ahmed, Shayan Taheri, Ive Weygers, Max Ortiz-Catalan

**Affiliations:** 1 Chalmers University, Chalmersplatsen 4, 412 96 Gothenburg, Sweden; 2 Aalto University, Espoo, Finland; 3 University of Erlangen, Nuremberg, Germany; 4 Prometei Pain Rehabilitation Center, Vinnytsia, Ukraine

**Keywords:** Gait analysis, Motion analysis, Prosthetic gait, Osseointegration, Transfemoral amputation gait, IMU motion capture, Inertial measurement unit, Joint kinematics, Motion capture validation, Orientation estimation algorithm

## Abstract

**Background:**

Systems that capture motion under laboratory conditions limit validity in real-world environments. Mobile motion capture solutions such as Inertial Measurement Units (IMUs) can progress our understanding of "real" human movement. IMU data must be validated in each application to interpret with clinical applicability; this is particularly true for diverse populations. Our IMU analysis method builds on the OpenSim IMU Inverse Kinematics toolkit integrating the Versatile Quaternion-based Filter and incorporates realistic constraints to the underlying biomechanical model. We validate our processing method against the reference standard optical motion capture in a case report with participants with transfemoral amputation fitted with a Percutaneous Osseointegrated Implant (POI) and without amputation walking over level ground. We hypothesis that by using this novel pipeline, we can validate IMU motion capture data, to a clinically acceptable degree.

**Results:**

Average RMSE (across all joints) between the two systems from the participant with a unilateral transfemoral amputation (TFA) on the amputated and the intact sides were 2.35° (IQR = 1.45°) and 3.59° (IQR = 2.00°) respectively. Equivalent results in the non-amputated participant were 2.26° (IQR = 1.08°). Joint level average RMSE between the two systems from the TFA ranged from 1.66° to 3.82° and from 1.21° to 5.46° in the non-amputated participant. In plane average RMSE between the two systems from the TFA ranged from 2.17° (coronal) to 3.91° (sagittal) and from 1.96° (transverse) to 2.32° (sagittal) in the non-amputated participant. Coefficients of Multiple Correlation (CMC) results between the two systems in the TFA ranged from 0.74 to > 0.99 and from 0.72 to > 0.99 in the non-amputated participant and resulted in ‘excellent’ similarity in each data set average, in every plane and at all joint levels. Normalized RMSE between the two systems from the TFA ranged from 3.40% (knee level) to 54.54% (pelvis level) and from 2.18% to 36.01% in the non-amputated participant.

**Conclusions:**

We offer a modular processing pipeline that enables the addition of extra layers, facilitates changes to the underlying biomechanical model, and can accept raw IMU data from any vendor. We successfully validate the pipeline using data, for the first time, from a TFA participant using a POI and have proved our hypothesis.

## Background

Joint kinematics offer an indication of deviation from unimpaired gait [1]. Clinical evaluation and gait pathology rehabilitation requires characterisation of gait (quality). Quantitative motion analysis is used to evaluate how an individual moves through space and is commonly performed using non-invasive, marker-based estimation techniques and optoelectronic motion capture systems have become the reference standard [2]. Despite applicability across industries, the space and cost of gait laboratories is often prohibitive and produces a vast data set which requires specialist knowledge, personnel and/or software to interpret and process. Moreover, gait quality is more accurately represented during real-world activities rather than under laboratory conditions that limit ecological validity [3]. Inertial Measurement Units (IMUs) are routinely used as mobile solutions to quantify motion [4] and can progress our understanding of "real" human movement and its implications for diverse populations such as those with lower limb amputations. IMUs are small, do not require a clear line of sight to collect data, and can be wirelessly affixed to an individual without obstructing movement. In both optoelectronic and inertial motion capture systems, the retroreflective markers or IMUs respectively feed to a biomechanical model where motions are transformed using rigid body kinematics into joint angles.

A challenge to the rigid body assumption, common to all marker based motion capture systems, is soft tissue movement which introduces motion artefacts [2, 5], a potential source of error which can increase average RMSE joint kinematics by up to 6° in gait [6]. In lower limb amputation, markers are typically affixed to the external surface of the prosthetic socket which introduces additional motion artefact due to socket pistoning (relative movement of residual limb and prosthetic socket). Several validation motion capture studies using both retroreflective markers and IMUs affix the retroreflective markers directly to the inertial markers in an effort to mitigate soft tissue (plus pistoning) artefacts on the measured joint angles [7–9]. However, these report a comparison between the measurement accuracy of the two *systems*, rather than between an optical or inertial gait *analysis*. Alternatively, placing the retroreflective markers on anatomical landmarks (the standard optical analysis method) [10–12] does not mitigate these motion artefacts but does provides a real-world methodological application of each system, e.g. the difference in the biomechanical models. Bony landmarks are not accessible along the length of the full residual limb of prosthetic socket users. However, an alternative to a prosthetic socket is a Percutaneous Osseointegrated Implant (POI), surgically connecting the skeleton to the artificial limb. Connecting to the user in this way enables retroreflective markers to be attached to all bone landmarks of the residual limb soft tissue, obviating motion artefacts from prosthetic socket pistoning.

The surgical procedure of POI minimizes residual limb soft tissue [13], in contrast to amputation surgery, often planned for prosthetic socket use i.e., retention of distal soft tissues for comfort [14]. This leads us to expect kinematic data from a POI user will be more like non amputated kinematic data compared to prosthetic socket users. In fact connection by POI results in a more symmetrical coronal plane gait pattern [15]. Furthermore, we expect this to reduce soft tissue and socket pistoning artefacts compared to prosthetic socket data when considering the lower limb markers. Several studies have validated and deployed IMU motion analyses in participants with lower limb amputation using prosthetic sockets [9, 10, 12, 16, 17], however the results cannot be generalised to include POI users. To the best of the authors knowledge there is no validation of a mobile motion capture system against the reference standard from real-world activities in users of POI. In order to progress our understanding of "real" human movement there is a clinical need to quantify gait quality from real-world activities. An ecological validity in this small but burgeoning population [18] will better inform POI design, surgical technique, and rehabilitation protocols.

There are several recent advances in orientation estimation algorithms and open-source toolboxes enabling such a validation. To generate a nuanced understanding of these advancements and their implications, we intend to provide an easy to use plug-and-play processing pipeline that, for the first time, integrates the current state-of-the-art in orientation estimation (Versatile Quaternion-based Filter) (VQF) [19] with the OpenSim IMU inverse kinematics (IK) toolkit (v4.4, Stanford University, USA) [20] applied to a constrained biomechanical model for joint angle estimation.

The Gait Deviation Index (GDI) [21] is one of many measures [22–24] of gait quality with a clinical application. We collected inertial and optical motion data from a participant with TFA implanted with a POI and one non-amputated participant. We compared estimated joint kinematic sets between optical and inertial motion capture systems and produced metrics that are the inputs to enable the calculation of GDI in future work. In doing so, we provide a validation for the inertial system and fill the knowledge gap on this population using our novel pipeline processing methods. Our hypothesis was that there would be no differences, at a clinically acceptable degree [25], between the motion capture systems.

## Methods

### Participants

TFA: A 27-year-old male with a right-sided unilateral, traumatic transfemoral amputation at aged 17, was fitted 2 years later with a POI (the Osseointegrated Prosthesis for the Rehabilitation of Amputees, OPRA), and took part in this study. The participant had good mobility (clinician assigned K Level 4) and used a microprocessor-controlled C-Leg connected to a mechanical carbon fibre Taleo foot (both Ottobock, Duderstadt, Germany). He was 1.88 m in height and weighed 75 kg with a BMI of 21.2 kg/m^2^.

Non amputated: A 48-year-old female with no amputations nor mobility restrictions participated in this study. She was 1.60 m in height and weighed 52 kg with a BMI of 20.3 kg/m^2^.

### Optoelectronic system (reference)

**Fig. 1:**
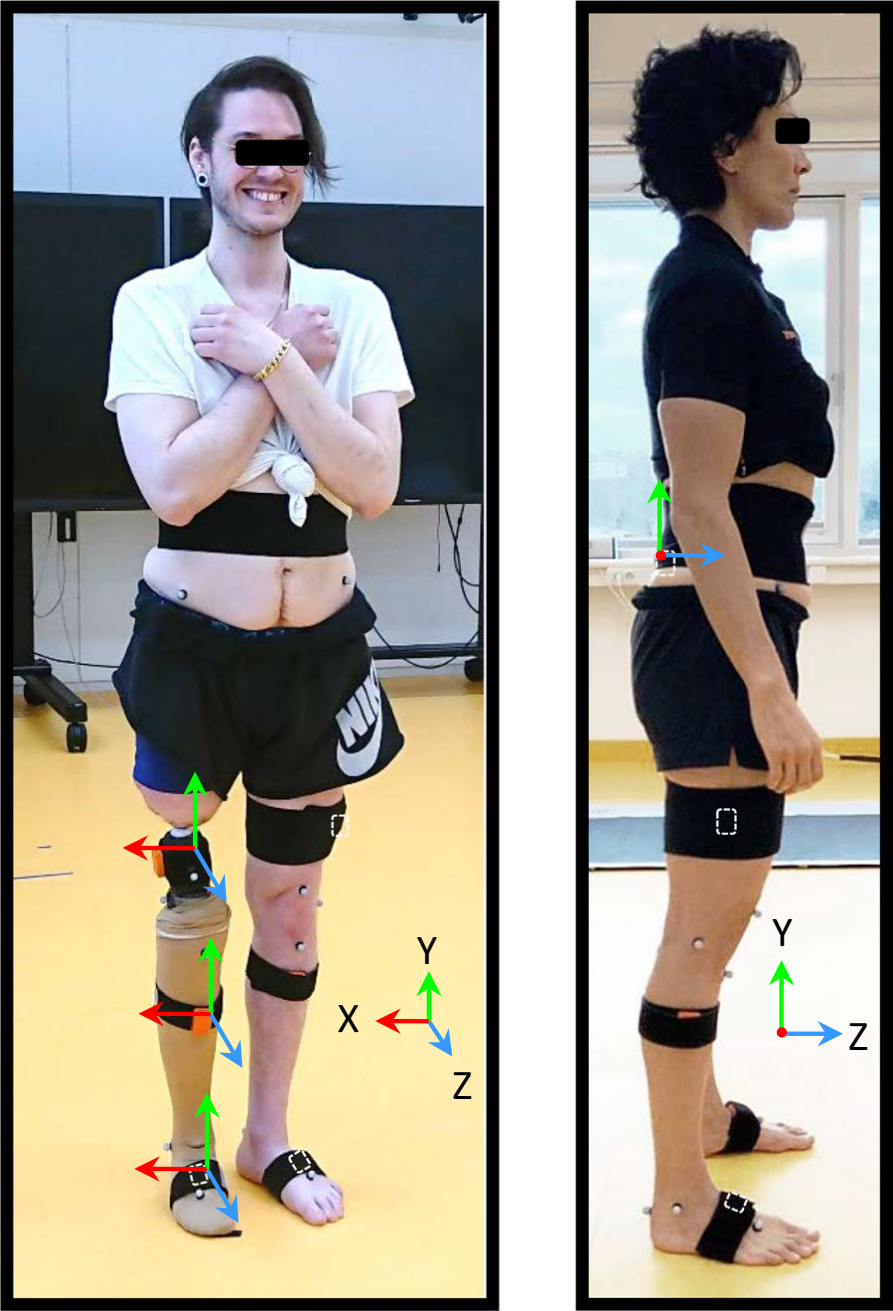
Retroreflective markers and IMUs (orange) mounted on stretch bands (black) while participants in calibration pose (note: IMUs hidden under stretch bands are indicated with a white dashed outline). The lower limb SUH marker set used: Sacrum, Anterior superior iliac spine, Lateral knee-joint line, Proximal to the superior border of the patella, Tibial tubercle, Heel, Lateral malleolus, and between the second and third Metatarsals. Laboratory and OpenSim biomechanical model coordinate systems shown

An infrared camera system (Qualisys AB, Gothenburg, Sweden) comprising 10 cameras was used in the optoelectronic motion capture. An experienced physiotherapist affixed 15 retroreflective markers to the participants using a modified version of the validated Sahlgrenska University Hospital (SUH) skin marker set [26]. For the TFA participant equivalent bony landmarks distal to the residual limb soft tissue on the artificial leg were used (Fig. 1). Prior to testing, calibration poses in a neutral standing position for both participants were recorded.

### Inertial motion capture system (estimate)

An Xsens Awinda system (Movella Inc., California, USA) was used to capture three-axis accelerometer and gyroscope, and magnetometer measurements from seven IMUs on each participant. Prior to data collection the IMUs were affixed with supplied stretch bands according to the manufacturer’s recommended procedure [27], ensuring not to occlude the retroreflective markers which require a clear line of sight to the cameras. The position of the IMUs were unrestricted (since each trial begins with a calibration pose; see experimental protocol).

### Data recording

Raw data were simultaneously recorded by the optoelectronic and inertial motion capture systems at the maximum manufacturer’s recommended rates of 240 Hz and 60 Hz, respectively. The start and end of each trial was marked by a trigger pulse generated by the Xsens system. Hardware synchronization was further validated and enhanced with software refinement via cross-correlation analysis and visual inspection.

### Experimental protocol

Prior to data collection, participants underwent several gait trials for 10 min to familiarize themselves with the gait conditions. Participant data collection sessions were not conducted at the same time. A total of seven data collection gait trials for the TFA participant and three trials for the non-amputated participant were recorded in two sessions. Each trial preceded by the inertial motion capture calibration pose. To ensure secure attachment of the IMUs to the foot segment, participants were asked to perform the trials barefoot.

Inertial motion capture system calibration started with a standing pose (feet parallel and hip-width apart, hip and knee directly in line above the feet, and a neutral spine alignment) followed by a six-meter straight line walk at a self-selected pace, a 180° turn and return walk repeatedly for 60 s.

Optoelectronic motion capture system calibration entailed one static reference recording while participants were standing aligned with the Z-axis of the global (lab) coordinate system (see Fig. 1).

## Data processing and analysis

### Optoelectronic system (reference)

Biomechanical models were created from participants static calibration pose in Visual3D Software (C-motion Inc., Germantown, USA), they had seven linked segments modeled as cylindrical rigid bodies with a mass and center of gravity. The models were scaled using participant height and segments were linked with six socket joints. Retroreflective marker trajectory data was processed and analyzed in Visual3D. A fourth-order 15 Hz Butterworth low-pass filter was applied, and a prediction approach was used to calculate hip, knee, and ankle joint centers. Segment joint angles were calculated and reported as the relative orientation of the distal segment with respect to the proximal segment described in the X-Y-Z Cardan sequence (i.e., flexion/extension, adduction/abduction angles, and internal/external rotation). The orientation of the pelvis was calculated with respect to the global (laboratory) frame and is described in the Z-Y-X Cardan sequence. A custom Python script (v3.11) was used to identify and mark heel strikes based on heel marker vertical position data to be used for stride segmentation in both systems.

### Inertial motion capture system (estimate)

The raw accelerometer, gyroscope and magnetometer data were wirelessly transmitted to a PC in real-time and processed using the VQF algorithm to obtain sensor orientations. For evaluation and error reporting purposes, the reference coordinate frames of the two systems were aligned by a transformation found by solving the robot-world/hand-eye calibration problem using the Kronecker product [28]. Next, the IMU data were processed with the OpenSim IMU IK toolkit to calculate joint kinematics [20]. The OpenSim IMU IK toolkit uses the first frame of a trial, assuming the participant is in the calibration pose, to compute sensor-to-segment calibration matrices. It then iterates through each time frame, finding joint angles that minimize orientation errors between the model and the measured orientations. We modified OpenSim's Gait 2354 physiological skeletal model [29] by simplifying the knee and ankle to a single hinge joint and locking the subtalar and metatarsophalangeal joints to represent the biomechanics of an artificial limb more accurately. The orientation of IMUs with respect to their underlying body segments was estimated in OpenSim using the initial pose of each trial. The time series obtained for each gait trial with both participants was parsed into individual strides using heel strike time marks. To minimize the influence of gait initiation (acceleration) or stopping (deceleration), we discarded the first and last full strides, as well as the strides during and immediately before and after each turn, as the change in direction during turns could introduce variability in gait dynamics. A total of 167 strides were included in the final comparison for the TFA participant and 111 strides for the non-amputated participant. For the calculation of foot progression angle we employed the methods in Wouda, Jaspar [30].

### Data processing and reporting

**Fig. 2:**
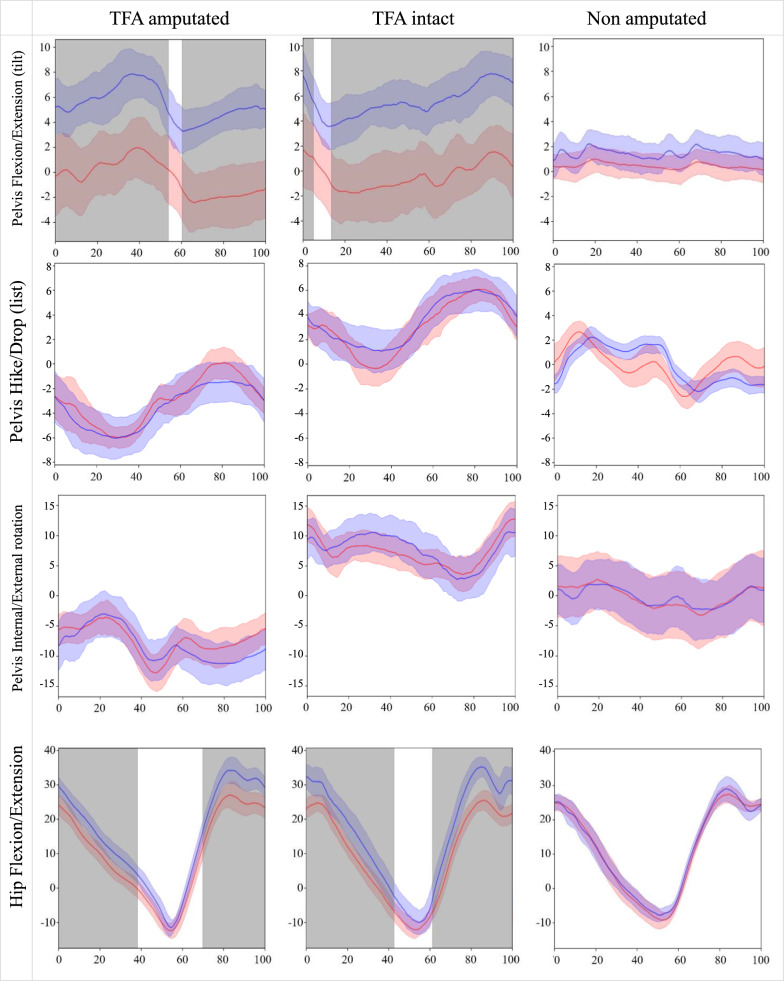

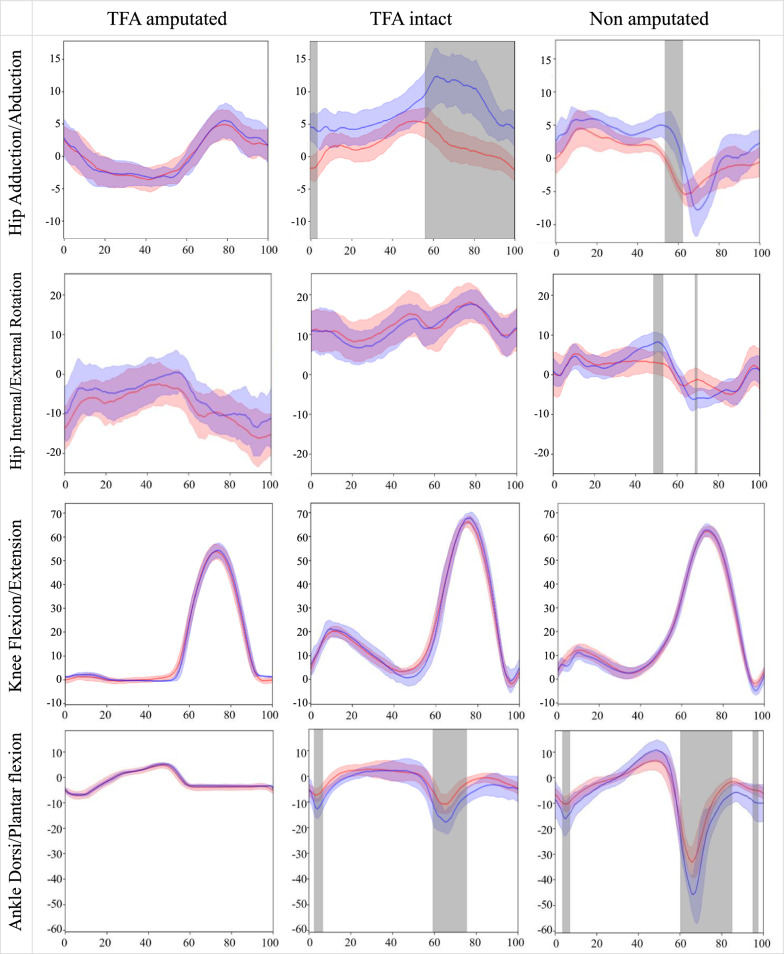
Joint angles averaged over all gait cycles. Blue = Inertial motion capture system ± IQR. Red = Optoelectronic system ± IQR. Shaded bands show regions where median RMSE > 5°. Gait cycle (%) horizontal axis is plotted against joint angles (°) on the vertical axis

For each joint, root mean square error (RMSE) and range of motion (ROM) were calculated for each stride. The normalized RMSE for each stride was calculated by dividing the RMSE by the ROM. Since the calculated RMSE, normalized (n)RMSE, and ROM values for each stride did not pass the Shapiro–Wilk test for normality we report joint angle central tendency (median) and Interquartile Range (IQR) estimates from both motion capture systems. We provide average ROM data for both systems and participants (Table 1) enabling calculation of the GDI (pelvis and hip in three planes, knee and ankle sagittal plane and foot progression angle). All non-amputated participant data is the sum of the right and left leg. We assess the similarity of the joint angle waveforms obtained through the two systems using Coefficients of Multiple Correlation (CMC) similar to Ferrari, Cutti [31] with a 95% CI calculated using non-parametric bootstrapping. CMC is reported where all values are real numbers i.e., from 0 to 1. Similarity of the joint angle waveforms was considered excellent if CMC > 0.75, fair-to-high if CMC 0.4–0.74, and poor if CMC < 0.39. We plotted the waveforms taking the n'th sample (n from 0 to 100) of each stride from each system, which composes the two observation sets (one set for each system). Then we took the RMSE between the two sets, if RMSE > 5°, we marked the sample and shaded it in Fig. 2. The absolute RMSE data was normalized (nRMSE) with respect to ROM obtained using the optical motion capture data on the same participant, similar to methods employed by Manz, Seifert [17].

**Table 1:** Average ROM (°) and foot progression angles across all gait cycles in TFA and non amputated participants

Joint/spatiotemporal measure	Optical						IMU					
	TFA amp	IQR	TFA intact	IQR	Without amputation	IQR	TFA amp	IQR	TFA intact	IQR	Without amputation	IQR
Pelvis Flexion/Extension (tilt)	4.87	1.11	4.31	1.20	1.69	0.55	4.81	1.18	4.74	1.18	2.39	0.70
Pelvis Hike/Drop (list)	6.13	1.27	6.62	1.31	5.39	0.76	4.64	1.22	4.89	0.95	4.55	0.60
Pelvis Internal/External rotation	9.68	2.24	9.96	1.86	7.25	2.65	9.51	2.62	9.15	1.85	6.23	2.49
Hip Flexion/Extension	39.42	2.75	39.32	1.75	37.01	1.33	45.48	2.89	47.41	2.28	37.03	2.33
Hip Adduction/Abduction	8.70	1.74	8.62	2.25	10.67	1.64	9.32	2.39	10.43	2.36	14.37	2.57
Hip Internal/External Rotation	10.83	2.37	15.20	3.63	11.72	2.42	11.21	2.55	15.53	4.21	15.00	2.74
Knee Flexion/Extension	54.56	3.16	68.30	2.07	64.64	2.64	54.85	3.08	69.88	5.38	68.09	1.59
Ankle Dorsi/Plantarflexion	12.14	0.71	14.80	2.85	39.94	5.97	12.06	0.61	21.60	4.49	57.33	10.48
Foot progression	7.28	3.70	14.74	4.38	3.02	21.02	6.93	4.05	15.59	5.42	4.29	19.42

## Results

ROM data average over all gait cycles between both motion capture systems for all participants is presented in Table 1.

### Average (combined) RMSE

TFA: Across all joints and spatiotemporal data, the amputated side was lower compared to the intact side; 2.35° (IQR = 1.45°) and 3.59° (IQR = 2.00°) respectively. There were no RMSEs above 5.98° on either side. Combined joint level average RMSE between the two systems ranged from 1.66° to 3.82° (foot progression and hip respectively). Combined in plane average RMSE ranged from 2.17° in the coronal plane to 3.91° in the sagittal plane.

Non amputated: Across all joints and spatiotemporal data average RMSE was 2.26° (IQR = 1.08°). There were no RMSEs above 5.46°. Joint level average RMSE between the two systems ranged from 1.21° to 5.46° (pelvis and ankle respectively). Average RMSE ranged from 1.96° in the transverse plane to 2.32° in the sagittal plane.

### CMC

TFA: Results (considering only real numbers) ranged from 0.74 (intact side pelvis internal/external rotation) to > 0.99 (knee flexion/extension on both sides) equating to from fair-to-high to an excellent similarity between the joint angle waveforms with an average of > 0.92 and > 0.89 on amputated and intact sides respectively (excellent similarity). Averaged in plane CMC data were lowest in transverse and highest in sagittal planes (0.81 and > 0.96 respectively). Averaged joint level results ranged from > 0.84 at the pelvis to > 0.99 at the knee (excellent similarity).

Non amputated: Results (considering only real numbers) ranged from 0.72 (pelvis hike/drop) to > 0.99 (knee and hip flexion/extension) with an average of > 0.88 demonstrating an excellent similarity between the joint angle waveforms. In plane CMC data was lowest in coronal and highest in sagittal planes (0.76 and > 0.98 respectively). Similar to the TFA results, averaged joint level results ranged from 0.79 at the pelvis to > 0.99 at the knee equating to excellent waveform similarity.

### nRMSE

TFA: There were outliers in pelvis flexion/extension on both legs (nRMSE amputated = 113.14% (IQR = 73.77), nRMSE intact = 132.48 (IQR = 75.59)), and intact side hip adduction/abduction (nRMSE = 69.37% (IQR = 26.35)) otherwise all nRMSE was < 27.69%. Joint level average nRMSE across all joints on the amputated side was lower compared to the intact side; 18.87% and 30.51% respectively. The combined joint level average nRMSE ranged from 3.40% at the knee joint level to 54.54% at the pelvis level. The combined in plane average nRMSE ranged from 22.59% in the transverse plane to 39.21% in the sagittal plane.

Non amputated: Although nRMSE in pelvis flexion/extension was higher than other non-amputated participant results (nRMSE = 66.86% (IQR = 61.45)) it was approximately half of that obtained from the TFA data in the same DOF. The joint level average nRMSE ranged from 2.18% at the knee joint level to 36.01% at the pelvis level. In plane average nRMSE ranged from 19.47% in the transverse plane to 27.18% in the coronal plane.

Waveform plots in Fig. 2 illustrate the congruency between both motion capture data sets where grey overlay denotes regions where median RMSE > 5°, a threshold in literature deemed acceptable for many applications [25].

## Discussion

Our novel signal processing pipeline has enabled the calculation of joint kinematics from raw IMU data for the first-time using POI participants. TFA combined average RMSE was 2.97° (2.35° and 3.59°; amputated and intact sides respectively) and 2.26° from the non-amputated participant data, is in line with non-amputated participant and TFA literature [10, 17, 20, 32–35]. Joint kinematic RMSE < 2° is considered to be excellent and < 5° is a clinically acceptable level of accuracy [25], errors greater than 5° could mislead clinical interpretation [36]. It is worth noting that these thresholds refer to the most common clinical situations and may not be suitable in all applications (for example in surgical decision making in children with Cerebral Palsy). Our non-amputated participant results had very few regions with an RMSE > 5° (grey shading on waveform plots) except for small sections in hip adduction/abduction, hip internal/external rotation, and ankle dorsi/plantar flexion just before and after the toe off phase of gait (~ 60% gait cycle). It has been previously suggested [10] that during toe off and heel contact gait phases one might expect the largest deviations between optical and IMU motion capture angles due to the difference in inertial properties of soft tissues. Extending this logic; since markers are placed on the residual soft tissues of POI users unlike the rigid material of a prosthetic socket of a conventional TFA, one might expect a greater difference between the intact and amputated legs of a socket user compared to the intact and amputated legs of a POI user. Comparing knee flexion/extension RMSE results from our study (1.59° amputated vs 2.66° intact, a multiple of 1.7) with the prosthetic socket data in Seel, Raisch [10] (0.71° amputated vs 3.30° intact, a multiple of 4.6) it seems that this could be the case although this should be followed up in future work with matched controls before deductions can be drawn.

The inertial motion capture system presented herein is subject to two primary sources of error when compared to the optoelectronic system reference standard: calibration errors and errors from information processing stages (i.e., IMU orientation and joint angle estimation algorithms). Calibration errors occur when the participant does not hold the calibration pose perfectly, leading to an erroneous sensor-to-segment calibration matrix. Such errors result in an offset in the measured joint angles, evident in Fig. 2 plots depicting pelvis and hip flexion/extension in the TFA participant. By eliminating the offset between the time series, we can compute RMSE values that are not influenced by calibration errors. Offset-corrected RMSE values (Table 2a in brackets) for the TFA data segments are all less than 2°, whereas some segments in the TFA-intact and non-amputated participant data still exhibit relatively high errors. Notably, the participant without amputation displays high RMSE values at the ankle despite offset correction. This could be attributed to two factors: the constrained biomechanical model offering a more accurate representation of the TFA participant ankle, and the absence of skin artefacts at both the foot and the shank, where the IMUs were installed, enhancing the accuracy of joint angle estimation. Furthermore, the lower ankle angle RMSE for the TFA-intact compared to non-amputated participant data can be explained by the fact that the range of ankle motion is nearly three times greater without amputation compared to TFA-intact.

**Table 2:** **a** Absolute measure of fit. **b** Absolute measure of fit

Median RMSE and IQR of joint angles between systems						
Joint / spatiotemporal measure	Absolute RMSE (°)					
	TFA Amputated	IQR	TFA Intact	IQR	Without amputation	IQR
Pelvis Flexion/Extension (tilt)	5.51 (0.84)	3.13 (0.21)	5.71 (0.83)	3.49 (0.25)	1.13 (0.39)	0.99 (0.11)
Pelvis Hike/Drop (list)	0.89 (0.61)	0.41 (0.29)	0.88 (0.66)	0.32 (0.34)	1.43 (1.38)	0.15 (0.12)
Pelvis Internal/External rotation	2.68 (1.94)	0.87 (0.41)	2.60 (1.87)	0.88 (0.48)	1.06 (0.73)	0.86 (0.12)
Hip Flexion/Extension	5.23 (1.64)	3.38 (0.32)	5.89 (2.43)	4.55 (0.30)	1.27 (0.99)	0.45 (0.38)
Hip Adduction/Abduction	1.03 (0.79)	0.70 (0.37)	5.98 (2.83)	2.15 (1.11)	2.97 (2.50)	0.57 (0.49)
Hip Internal/External Rotation	1.95 (1.03)	1.36 (0.32)	2.82 (1.59)	1.73 (0.51)	2.85 (2.44)	1.23 (0.63)
Knee Flexion/Extension	1.59 (1.28)	0.48 (0.50)	2.66 (2.27)	0.55 (0.36)	1.41 (1.33)	0.21 (0.23)
Ankle Dorsi/Plantarflexion	0.78 (0.34)	0.73 (0.09)	3.93 (2.35)	1.88 (0.42)	5.46 (4.25)	2.48 (1.70)
Foot progression angle	1.48	2.01	1.84	2.46	2.73	2.80
Average	2.35	1.45	3.59	2.00	2.26	1.08

Plane and joint level median RMSE and radar plot between systems						
Absolute RMSE (°)						
		TFA Amputated	TFA Intact	Combined	Without amputation	
Plane	Sagittal	3.28	4.55	3.91	2.32	
	Coronal	0.96	3.39	2.17	2.20	
	Transverse	2.32	2.71	2.51	1.96	
Joint level	Pelvis	3.03	3.06	3.05	1.21	
	Hip	2.74	4.90	3.82	2.36	
	Knee	1.59	2.66	2.13	1.41	
	Ankle	0.78	3.93	2.36	5.46	
	Foot	1.48	1.84	1.66	2.73	

Numbers in brackets reflect bias-corrected median and IQR RMSE values

Radar plot key: Green = TFA amputated side. Purple = TFA intact side. Black = TFA combined. Orange = without amputation

Soft tissue artefacts influence all planes of motion, with the sagittal and transverse planes often generating the most and least reliable motion data respectively (due also in part to locating an accurate hip joint center) [5, 37, 38]. In both participants we observed the highest CMC in the sagittal planes and the TFA data produced the lowest CMC in the transverse plane, reflecting the literature in terms of similarity of joint angle waveforms. Further work with more TFA participants using POI would be a useful follow on from this study to confirm that it is echoed amongst this population. We obtained similar absolute RMSE at magnitude in hip flexion/extension to Manz, Seifert [17] who used TFA participants with prosthetic socket connections. Moreover, at the knee and ankle level our absolute RMSE results match an even greater proportion of the literature [10, 17, 39]. The in plane combined TFA and without amputation RMSEs resulted in a clinically acceptable level of accuracy at < 4.0° and < 2.5° respectively, with excellent average joint angle waveform similarity in each population (> 0.92, > 0.89, and > 0.88 amputated, intact and without amputation respectively) (Table 3).

**Table 3:** **a** Relative measure of fit – correlation of waveforms. **b** Relative measure of fit–correlation of waveforms

CMC between systems						
Joint / spatiotemporal measure	CMC					
	TFA Amputated	CI	TFA Intact	CI	Without amputation	CI
Pelvis Flexion/Extension (tilt)	nan	–	nan	–	nan	–
Pelvis Hike/Drop (list)	0.93	(0.93–0.94)	0.94	(0.94–0.95)	0.72	(0.67–0.77)
Pelvis Internal/External rotation	0.76	(0.73–0.79)	0.74	(0.71–0.78)	0.86	(0.85–0.87)
Hip Flexion/Extension	0.96	(0.95–0.96)	0.94	(0.94–0.95)	> 0.99	(1.00–1.00)
Hip Adduction/Abduction	0.96	(0.96–0.96)	nan	–	0.80	(0.77–0.84)
Hip Internal/External Rotation	0.87	(0.86–0.89)	0.88	(0.87–0.90)	0.83	(0.82–0.85)
Knee Flexion/Extension	> 0.99	(1.00–1.00)	> 0.99	(1.00 -1.00)	> 0.99	(1.00–1.00)
Ankle Dorsi/Plantarflexion	0.98	(0.98–0.98)	0.86	(0.84–0.87)	0.95	(0.94–0.95)
Foot progression angle	–	–	–	–	–	–
Average	> 0.92		> 0.89		> 0.88	

Plane and joint level CMC between systems					
		CMC			
		TFA Amputated	TFA Intact	Combined	Without amputation
Plane	Sagittal	0.98	0.93	0.96	> 0.98
	Coronal	0.95	0.94	0.94	0.76
	Transverse	0.82	0.81	0.81	0.85
Joint level	Pelvis	0.85	0.84	0.84	0.79
	Hip	0.93	0.91	0.92	0.82
	Knee	> 0.99	> 0.99	> 0.99	> 0.99
	Ankle	0.98	0.86	0.92	0.95

Real numbers are reported for CMC (from 0 to 1)

Most of the literature focuses on absolute RMSE and we consider that normalizing against joint ROM has been somewhat overlooked. The value of this added data dimension is highlighted in the radar plots in Tables 2b and 4b where absolute RMSE averages depict the ankle and hip plane levels to be the most divergent between systems (largest errors). In fact, when considered as a ratio (nRMSE) of the overall ROM, the pelvis level ubiquitously results in the greatest errors. This is driven predominantly by our result in pelvis flexion/extension RMSE (5.51° amputated and 5.71° intact) since ROM at the same joint level and degree of freedom is 4.87° and 4.31° respectively. Otherwise our nRMSE results were similar to the literature with and without lower limb amputations; Manz, Seifert [17] obtained a nRMSE of 0.75% and 11.67% at the knee and hip level respectively in TFAs. Our TFA amputated side equivalents were 2.91% and 14.37% respectively. Our methods enabled the placement of IMUs in any position on each body segment for data collection, conversely Manz, Seifert [17] embedded IMU sensors inside the prosthetic leg and so the relative orientation of IMUs with respect to segments was known. This obviates the need to perform sensor-to-segment calibration and so drastically improves results. Teufl, Lorenz [40] observed an average joint angle nRMSE < 7%; our TFA data was under this threshold in three out of eight results (knee on both legs and ankle on the amputated side). Average joint angle nRMSE of 13.2%–29.3% were obtained by Lim, Kim [41], our equivalent results were within a similar range in the TFA amputated and non-amputated participant data (25.97% and 22.43% respectively) but outside this range in the TFA intact side (38.83%). It is likely that the increased accuracy we obtained on the amputated TFA leg compared to the intact leg were due to soft tissue artefacts. The waveform similarity data has resulted in ‘excellent’ similarity in each data set average, in every plane and at all joint levels.

**Table 4:** **a** Relative measure of fit. **b** Relative measure of fit

nRMSE and IQR of joint angles between systems						
Joint / spatiotemporal measure	nRMSE (%)					
	TFA Amputated	IQR	TFA Intact	IQR	Without amputation	IQR
Pelvis Flexion/Extension (tilt)	113.14	73.77	132.48	75.59	66.86	61.45
Pelvis Hike/Drop (list)	14.52	6.99	13.29	4.80	26.53	4.86
Pelvis Internal/External rotation	27.69	12.23	26.10	11.11	14.62	14.10
Hip Flexion/Extension	13.27	8.15	14.98	12.14	3.43	1.29
Hip Adduction/Abduction	11.84	7.36	69.37	26.35	27.84	8.22
Hip Internal/External Rotation	18.01	13.51	18.55	12.80	24.32	11.35
Knee Flexion/Extension	2.91	0.94	3.89	0.88	2.18	0.37
Ankle Dorsi/Plantarflexion	6.43	5.72	32.59	11.56	13.67	4.80
Foot progression angle	–	–	–	–	–	–
Average	25.97	16.08	38.83	19.40	22.43	13.31

Plane and joint level nRMSE and radar plot between systems						
nRMSE (%)						
		TFA Amputated	TFA Intact	Combined	Without amputation	
Plane	Sagittal	33.94	44.48	39.21	21.54	
	Coronal	13.18	41.33	27.26	27.18	
	Transverse	22.85	22.33	22.59	19.47	
Joint level	Pelvis	51.78	57.29	54.54	36.01	
	Hip	14.37	34.30	24.34	18.53	
	Knee	2.91	3.89	3.40	2.18	
	Ankle	6.43	26.55	16.49	13.67	

Radar plot key: Green = TFA amputated side. Purple = TFA intact side. Black = TFA combined. Orange = without amputation

We acknowledge there was a potential for increased error in our methods arising from sensor-to-segment calibration error in addition to not mounting the retroreflective markers on the IMUs and not having a rigid prosthetic socket to attach sensors to. As a result, however we have been able to offer an unrestricted IMU placement and our methods have been sophisticated enough to produce results that are similar to the literature. We note that this may have been partly influenced by the soft tissue surgery entailed in POI fixation and that our participants did not have a high BMI. Our processing pipeline enables the calculation of GDI, enabling the classification of gait deviation which can potentially progress our understanding of 'real' human movement and its implications for diverse populations. Finally, joint kinematics can provide valuable information that will better inform POI design, surgical technique, and rehabilitation protocols [42].

## Strengths and limitations and future work

All modelling is a best approximation, and despite the modifications made to the OpenSim model, inaccuracies still remain in both models, such as participant geometry, calibration pose accuracy, and the imperfect representation of joints as hinge-like structures during gait. Typically, gait compensation techniques in TFA lead to reduced thorax-pelvis rotational ROM and pelvic obliquity throughout the gait cycle [43, 44]. Despite POI purporting more symmetrical coronal plane gait, these deviations were not accounted for. Other potential sources of error include high intra and inter participant variability in the assessment of anatomical landmarks and joint center’s [5, 35, 37, 38]. Employing anatomical calibration procedures can mitigate this artefact to some extent however, the kinaesthetic ability of participants to reproduce calibration postures and motion is a potential source of error and subject effects may be generated. It is worth noting that there may also be an innate source of experimental error due to the weight and attachment band of the IMUs which may dampen higher-frequency perturbations that would otherwise affect the optical motion capture markers. It should be noted that real world conditions may influence results and that there may be some unknowns associated with longer data collection periods.

Another notable source of inaccuracy in IMU motion capture systems arises from drift error in IMU measurements. In this study, we performed static single-pose calibration before each trial to minimize the effect of drift error between trials. While this calibration method has shown high repeatability [45], performing calibration as frequently may not be feasible in real-world applications. However, previous studies have demonstrated that using model-based inverse kinematics can significantly reduce IMU drift error in joint angle estimation over extended recording periods [20]. Additionally, the VQF algorithm has been shown to be highly effective in eliminating drift by accurately estimating and compensating for gyroscope bias. Therefore, we consider it an important next step to evaluate the introduced pipeline in a real-world population-specific application with less frequent calibration.

The strength of this study is in the easy-to-use plug-and-play processing pipeline, the ability to freely place the IMUs and our novel processing method. These methods have generated a clinically acceptable [25], level of accuracy in data sets from two completely different participants in terms of mobility, kinematics and anthropomorphic features demonstrating the strength of these processing methods. We obtained 167 strides for the comparison; more strides from additional participants might allow us to perform interparticipant tests of significance.

Previous validations on participants with transfemoral amputation using prosthetic sockets has presumed that the socket mitigated effects of soft tissue artefacts on results [10]. We have offered a validated processing pipeline by which to calculate these effects since POI removes the influence of socket pistoning. Further work might exploit the advantages that POI offers whereby participants can ambulate either using adapted prosthetic sockets or as intended, via their POI and a direct controlled study could be undertaken. Furthermore, it has been shown recently that POI markers affixed directly to the exoprosthetic portion rather than skin markers representing the same segment are a more accurate representation of the true femoral movement [46], this might aid in the development of accuracy in future work.

## Conclusions

Our signal processing pipeline works out of the box and has a customizable extensible architecture enabling the easy addition of extra layers, such as more sophisticated calibration routines. The pipeline is based on the OpenSim IMU IK toolkit, extended to incorporate the highly customizable VQF algorithm. This addition allows the pipeline to directly accept raw IMU data from any vendor. Users can modify the underlying OpenSim biomechanical model, for example, by adding more realistic constraints for a specific target group, changing joint types, and use in other limb level amputation applications. We have successfully demonstrated a clinically acceptable [25] level of accuracy between an optical and inertial motion capture system to support our hypothesis using our novel processing pipeline applied to participants with TFA using POI and without amputation data sets. This achievement signifies an important step towards real-world evaluation of gait quality beyond laboratory settings, with application in diverse populations.

## Data Availability

The datasets generated and/or analyzed during the current study are available in the Github repository, implemented in Python under the MIT license at https://github.com/Motion-Capture/Osseointegrated-IMU.

## References

[1] Kuo AD, Donelan JM. Dynamic principles of gait and their clinical implications. Phys Ther. 2010;90(2):157–74. 20023002 10.2522/ptj.20090125 PMC2816028

[2] Colyer SL, Evans M, Cosker DP, Salo AI. A review of the evolution of vision-based motion analysis and the integration of advanced computer vision methods towards developing a markerless system. Sports Med-Open. 2018;4(1):1–15. 29869300 10.1186/s40798-018-0139-y PMC5986692

[3] Cutti AG, Raggi M, Andreoni G, Sacchetti R. Clinical gait analysis for amputees: innovation wishlist and the perspectives offered by the outwalk protocol. G Ital Med Lav Ergon. 2015;37(3):45–8. 26731957

[4] Uhlrich SD, Falisse A, Kidziński Ł, Muccini J, Ko M, Chaudhari AS, et al. OpenCap: 3D human movement dynamics from smartphone videos. BioRxiv. 2022;98:109451. 10.1371/journal.pcbi.1011462 PMC10586693 37856442

[5] Leardini A, Chiari L, Croce UD, Cappozzo A. Human movement analysis using stereophotogrammetry: Part 3. Soft tissue artifact assessment and compensation. Gait Posture. 2005;21(2):212–25. 15639400 10.1016/j.gaitpost.2004.05.002

[6] Akbarshahi M, Schache AG, Fernandez JW, Baker R, Banks S, Pandy MG. Non-invasive assessment of soft-tissue artifact and its effect on knee joint kinematics during functional activity. J Biomech. 2010;43(7):1292–301. 20206357 10.1016/j.jbiomech.2010.01.002

[7] Zhang J-T, Novak AC, Brouwer B, Li Q. Concurrent validation of Xsens MVN measurement of lower limb joint angular kinematics. Physiol Meas. 2013;34(8):N63. 23893094 10.1088/0967-3334/34/8/N63

[8] Schepers M, Giuberti M, Bellusci G. Xsens MVN: consistent tracking of human motion using inertial sensing. Xsens Technol. 2018;1(8):1–8.

[9] Rattanakoch J, Samala M, Limroongreungrat W, Guerra G, Tharawadeepimuk K, Nanbancha A, et al. Validity and reliability of inertial measurement unit (IMU)-derived 3D joint kinematics in persons wearing transtibial prosthesis. Sensors. 2023;23(3):1738. 36772783 10.3390/s23031738 PMC9920655

[10] Seel T, Raisch J, Schauer T. IMU-based joint angle measurement for gait analysis. Sensors. 2014;14(4):6891–909. 24743160 10.3390/s140406891 PMC4029684

[11] Takeda R, Tadano S, Natorigawa A, Todoh M, Yoshinari S. Gait posture estimation using wearable acceleration and gyro sensors. J Biomech. 2009;42(15):2486–94. 19682694 10.1016/j.jbiomech.2009.07.016

[12] Finco M, Patterson RM, Moudy SC. A pilot case series for concurrent validation of inertial measurement units to motion capture in individuals who use unilateral lower-limb prostheses. J Rehabil Assist Technol Eng. 2023;10:20556683231182320. 37441370 10.1177/20556683231182322 PMC10334000

[13] Marano AA, Modiri O, Rozbruch SR, Otterburn DM. Soft tissue contouring at the time of osseointegrated implant reconstruction for lower extremity amputation. Ann Plast Surg. 2020;85(S1):S33–6. 32187066 10.1097/SAP.0000000000002329

[14] Schnur D, Meier RH. Amputation surgery. Phys Med Rehabil Clin. 2014;25(1):35–43. 10.1016/j.pmr.2013.09.013 24287238

[15] Darter BJ, Syrett ED, Foreman KB, Kubiak E, Sinclair S. Changes in frontal plane kinematics over 12-months in individuals with the Percutaneous Osseointegrated Prosthesis (POP). PLoS ONE. 2023;18(2): e0281339. 36812173 10.1371/journal.pone.0281339 PMC9946262

[16] Clemens S, Kim KJ, Gailey R, Kirk-Sanchez N, Kristal A, Gaunaurd I. Inertial sensor-based measures of gait symmetry and repeatability in people with unilateral lower limb amputation. Clin Biomech. 2020;72:102–7. 10.1016/j.clinbiomech.2019.12.007 31862603

[17] Manz S, Seifert D, Altenburg B, Schmalz T, Dosen S, Gonzalez-Vargas J. Using embedded prosthesis sensors for clinical gait analyses in people with lower limb amputation: a feasibility study. Clin Biomech. 2023;106: 105988. 10.1016/j.clinbiomech.2023.105988 37230008

[18] Hebert JS, Rehani M, Stiegelmar R. Osseointegration for lower-limb amputation: a systematic review of clinical outcomes. JBJS reviews. 2017;5(10): e10. 29087966 10.2106/JBJS.RVW.17.00037

[19] Laidig D, Seel T. VQF: Highly accurate IMU orientation estimation with bias estimation and magnetic disturbance rejection. Information Fusion. 2023;91:187–204. 10.1016/j.inffus.2022.10.014

[20] Al Borno M, O’Day J, Ibarra V, Dunne J, Seth A, Habib A, et al. OpenSense: an open-source toolbox for inertial-measurement-unit-based measurement of lower extremity kinematics over long durations. J Neuroeng Rehabil. 2022;19(1):22. 35184727 10.1186/s12984-022-01001-x PMC8859896

[21] Schwartz MH, Rozumalski A. The gait deviation index: a new comprehensive index of gait pathology. Gait Posture. 2008;28(3):351–7. 18565753 10.1016/j.gaitpost.2008.05.001

[22] Schutte LM, Narayanan U, Stout JL, Selber P, Gage JR, Schwartz MH. An index for quantifying deviations from normal gait. Gait Posture. 2000;11(1):25–31. 10664482 10.1016/S0966-6362(99)00047-8

[23] Kark L, Vickers D, McIntosh A, Simmons A. Use of gait summary measures with lower limb amputees. Gait Posture. 2012;35(2):238–43. 22000790 10.1016/j.gaitpost.2011.09.013

[24] Baker R, McGinley JL, Schwartz MH, Beynon S, Rozumalski A, Graham HK, et al. The gait profile score and movement analysis profile. Gait Posture. 2009;30(3):265–9. 19632117 10.1016/j.gaitpost.2009.05.020

[25] McGinley JL, Baker R, Wolfe R, Morris ME. The reliability of three-dimensional kinematic gait measurements: a systematic review. Gait Posture. 2009;29(3):360–9. 19013070 10.1016/j.gaitpost.2008.09.003

[26] Tranberg R, Saari T, Zügner R, Kärrholm J. Simultaneous measurements of knee motion using an optical tracking system and radiostereometric analysis (RSA). Acta Orthop. 2011;82(2):171–6. 21463221 10.3109/17453674.2011.570675 PMC3235287

[27] Movella. Sensor Placement in Xsens Awinda System 2022. https://base.xsens.com/s/article/Sensor-Placement-in-Xsens-Awinda-System.

[28] Shah M. Solving the robot-world/hand-eye calibration problem using the kronecker product. J Mech Robot. 2013. 10.1115/1.4024473. 10.1115/1.4024473

[29] Delp SL, Loan JP, Hoy MG, Zajac FE, Topp EL, Rosen JM. An interactive graphics-based model of the lower extremity to study orthopaedic surgical procedures. IEEE Trans Biomed Eng. 1990;37(8):757–67. 2210784 10.1109/10.102791

[30] Wouda FJ, Jaspar SL, Harlaar J, van Beijnum BJF, Veltink PH. Foot progression angle estimation using a single foot-worn inertial sensor. J NeuroEngineering Rehabil. 2021;18:1–10. 10.1186/s12984-021-00816-4 PMC7888122 33596942

[31] Ferrari A, Cutti AG, Cappello A. A new formulation of the coefficient of multiple correlation to assess the similarity of waveforms measured synchronously by different motion analysis protocols. Gait Posture. 2010;31(4):540–2. 20303272 10.1016/j.gaitpost.2010.02.009

[32] Slade P, Habib A, Hicks JL, Delp SL. An open-source and wearable system for measuring 3D human motion in real-time. IEEE Trans Biomed Eng. 2022;69(2):678–88. 34383640 10.1109/TBME.2021.3103201 PMC8792207

[33] Adamowicz L, Gurchiek RD, Ferri J, Ursiny AT, Fiorentino N, McGinnis RS. Validation of novel relative orientation and inertial sensor-to-segment alignment algorithms for estimating 3D Hip joint angles. Sensors. 2019;19(23):5143. 31771263 10.3390/s19235143 PMC6929122

[34] Cordillet S, Bideau N, Bideau B, Nicolas G. Estimation of 3D knee joint angles during cycling using inertial sensors: accuracy of a novel sensor-to-segment calibration procedure based on pedaling motion. Sensors. 2019;19(11):2474. 31151200 10.3390/s19112474 PMC6603641

[35] McGrath T, Stirling L. Body-worn IMU-based human hip and knee kinematics estimation during treadmill walking. Sensors. 2022;22(7):2544. 35408159 10.3390/s22072544 PMC9003309

[36] Cuesta-Vargas AI, Galán-Mercant A, Williams JM. The use of inertial sensors system for human motion analysis. Phys Ther Rev. 2010;15(6):462–73. 23565045 10.1179/1743288X11Y.0000000006 PMC3566464

[37] Kainz H, Carty CP, Modenese L, Boyd RN, Lloyd DG. Estimation of the hip joint centre in human motion analysis: a systematic review. Clin Biomech. 2015;30(4):319–29. 10.1016/j.clinbiomech.2015.02.005 25753697

[38] Tsushima H, Morris ME, McGinley J. Test-retest reliability and inter-tester reliability of kinematic data from a three-dimensional gait analysis system. J Jpn Phys Ther Assoc. 2003;6(1):9–17. 25792928 10.1298/jjpta.6.9 PMC4316510

[39] Sharif Bidabadi S, Murray I, Lee GYF. Validation of foot pitch angle estimation using inertial measurement unit against marker-based optical 3D motion capture system. Biomed Eng Lett. 2018;8:283–90. 30603212 10.1007/s13534-018-0072-5 PMC6208541

[40] Teufl W, Lorenz M, Miezal M, Taetz B, Fröhlich M, Bleser G. Towards inertial sensor based mobile gait analysis: event-detection and spatio-temporal parameters. Sensors. 2019;19(1):38. 10.3390/s19010038 PMC6339047 30583508

[41] Lim H, Kim B, Park S. Prediction of lower limb kinetics and kinematics during walking by a single IMU on the lower back using machine learning. Sensors. 2019;20(1):130. 31878224 10.3390/s20010130 PMC6982819

[42] Ahmed K, Pendegrass C, Aston W, Blunn G. Radiographic Evidence of Bone Changes Around Intraosseous Transcutaneous Amputation Prosthesis: An 11-Year Retrospective Cohort Study. JPO: J Prosthet Ortho 2024:10-97.

[43] Esquenazi A. Gait analysis in lower-limb amputation and prosthetic rehabilitation. Phys Med Rehabil Clin. 2014;25(1):153–67. 10.1016/j.pmr.2013.09.006 24287245

[44] Carse B, Scott H, Brady L, Colvin J. A characterisation of established unilateral transfemoral amputee gait using 3D kinematics, kinetics and oxygen consumption measures. Gait Posture. 2020;75:98–104. 31645007 10.1016/j.gaitpost.2019.09.029

[45] Berner K, Cockcroft J, Morris LD, Louw Q. Concurrent validity and within-session reliability of gait kinematics measured using an inertial motion capture system with repeated calibration. J Bodyw Mov Ther. 2020;24(4):251–60. 33218520 10.1016/j.jbmt.2020.06.008

[46] Ravari R, Lewicke J, Vette AH, Hebert JS. Differences in angular kinematics when using thigh, implant, or medial knee markers in osseointegrated transfemoral prosthetic gait. Clin Biomech. 2023;105: 105976. 10.1016/j.clinbiomech.2023.105976 37127007

